# Medicinal Plants as Sources of Active Molecules Against COVID-19

**DOI:** 10.3389/fphar.2020.01189

**Published:** 2020-08-07

**Authors:** Bachir Benarba, Atanasio Pandiella

**Affiliations:** ^1^ Laboratory Research on Biological Systems and Geomatics, Faculty of Nature and Life Sciences, University of Mascara, Mascara, Algeria; ^2^ Instituto de Biología Molecular y Celular del Cáncer, CSIC-IBSAL-Universidad de Salamanca, Salamanca, Spain

**Keywords:** Severe Acute Respiratory Syndrome-related Coronavirus 2 (SARS-CoV-2), Coronavirus Disease 2019 (COVID-19), plants, natural products, ACE2

## Abstract

The Severe Acute Respiratory Syndrome-related Coronavirus 2 (SARS-CoV-2) or novel coronavirus (COVID-19) infection has been declared world pandemic causing a worrisome number of deaths, especially among vulnerable citizens, in 209 countries around the world. Although several therapeutic molecules are being tested, no effective vaccines or specific treatments have been developed. Since the COVID-19 outbreak, different traditional herbal medicines with promising results have been used alone or in combination with conventional drugs to treat infected patients. Here, we review the recent findings regarding the use of natural products to prevent or treat COVID-19 infection. Furthermore, the mechanisms responsible for this preventive or therapeutic effect are discussed. We conducted literature research using PubMed, Google Scholar, Scopus, and WHO website. Dissertations and theses were not considered. Only the situation reports edited by the WHO were included. The different herbal products (extracts) and purified molecules may exert their anti-SARS-CoV-2 actions by direct inhibition of the virus replication or entry. Interestingly, some products may block the ACE-2 receptor or the serine protease TMPRRS2 required by SARS-CoV-2 to infect human cells. In addition, natural products were shown to inhibit the SARS-CoV-2 life-cycle related proteins such as papain-like or chymotrypsin-like proteases. In conclusion, we suggest that natural products could be used alone or in combination as alternative medicines to treat/prevent COVID-19 infection. Moreover, their structures may offer clues for the development of anti-SARS-CoV-2 drugs.

## Introduction

The Severe Acute Respiratory Syndrome-related Coronavirus 2 or novel coronavirus (COVID-19) infection has been declared world pandemic resulting in thousands of deaths in 216 countries around the world. The disease appeared in late December 2019 in Wuhan (China) as a result of zoonotic transmission (Mackenzie and Smith, 2020). Actually, the Severe Acute Respiratory Syndrome-related Coronavirus 2 (SARS-CoV-2) was shown to share 96% of the genomic identity with the related bat coronavirus (Zhou et al., 2020). Moreover, the SARS-CoV-2 genome was found to be 91.02% identical to that of the Pangolin-CoV, raising the possibility that the latter acted as an intermediate zoonotic host between bats and humans (Zhang T. et al., 2020). Till now, no vaccines or specific treatments for SARS-CoV-2 have been developed, although extraordinary efforts are being made (Amanat and Krammer, 2020). Some therapeutic approaches have been suggested such as nucleoside analogs, Remdesivir, anti-inflammatory drugs or Lopinavir/Ritonavir to treat COVID-19. At present, more than 200 clinical trials, some of them analyzing these drugs or others, have been registered in clinicaltrials.gov. Nevertheless, the clinical usefulness of these drugs against COVID-19 infection remains unclear (Li et al., 2020).

Herbal traditional medicines have been used in China since the first days of the COVID-19 outbreak. Indeed, these traditional medicines were shown to result in the recovery of 90% of the 214 patients treated (Hong-Zhi et al., 2020). Furthermore, some traditional herbal medicines prevented SARS-CoV-2 infection of healthy persons and improved the health state of patients with mild or severe symptoms (Hong-Zhi et al., 2020). Similar promising results were reported in Zhejiang Province – China (Xu K. et al., 2020). Chinese traditional medicines known as Shu Feng Jie Du and Lianhuaqingwen have been recommended due to their demonstrated efficacy against previous influenza A (H1N1) or SARS-CoV-1 (Lu, 2020). A group of experts from the Zhongnan Hospital of Wuhan University included the use of traditional medicines in the guidelines for the treatment and prevention of COVID-19. Several methods using medicinal plants were recommended for the prevention of COVID-19. Moreover, to treat the disease, the experts recommended the use of different herbal mixtures according to the disease-stage (Jin et al., 2020).

This review focuses on the possible uses of herbal traditional medicines and natural products in the prevention and treatment of COVID-19 infection (**Table 1**).

**Table 1:** Natural products tested against coronaviruses.

Plant (Family/part)	Product	Model/Strains	Inhibitory assay	Dosage/duration	Control	Effects	Reference
*Alnus japonica* (Thunb.) Steud. (Betulaceae/bark)	Hirsutenone (Ethanol extract)	*In vitro* SARS-CoV- PLpro	FRET	0–200 µM/60 min	Curcumin (viral protease inhibitor)	A dose-dependent inhibition of the SARS-CoV- PLpro activity (IC_50_ = 4.1 ± 0.3 µM while that of curcumin was 5.7 ± 0.3 µM)	Park et al., 2012b
*Angelica keiskei* (Miq.) Koidz (Umbelliferae/leaves)	Xanthoangelol E (Ethanol extract)	*In vitro* SARS-CoV- PLpro SARS-CoV- 3CL(pro)	FRET Cell-based cis-cleavage inhibition assay	0, 12.5, 25, 50 µM	NS	A dose-dependent inhibition of SARS-CoV- PLpro activity (IC_50_ = 1.2 ± 0.4 µM) A dose-dependent inhibition of SARS-CoV- 3CL(pro) activity (IC_50_ = 11.4 ± 1.4 µM)	Park et al., 2016
*Aglaia perviridis* Hiern (Meliaceae/whole)	Myricetin	*In vitro* Angiotensin converting enzyme (ACE) from rabbit lung	FRET	0.01–10 µM	NS	Inhibition of the SARS-CoV helicase by affecting the ATPase activity (IC_50_ = 2.71 ± 0.19 μM)	Yu et al., 2012
*Cibotium barometz* (L.) J.Sm. (Dicksoniaceae/rhizomes)	Ethanol and methanol extracts	*In vitro* SARS-CoV virus propagated in Vero E6 cells	Cytopathic effect inhibition ELISA FRET	0, 25, 50, 100, and 200 µg/ml	Vero E6 cells without extracts (negative control) SARS-CoV-infected Vero E6 cells (positive control) Valinomycin (reference antiviral standard)	Both extracts inhibited the SARS-CoV- replication at concentrations of 25 and 200 μg/ml. EC_50_ were found to be 8.42 and ≥10 µg/ml, respectively.	Wen et al., 2011
Cullen corylifolium (L.) Medik. (Leguminosae/seeds)	Psoralidin (Ethanol extract)	*In vitro* SARS-CoV- PLpro	Fluorogenic assay	0–100µM	NS	Inhibition of SARS-CoV PLpro in a dose-depenedent manner with IC_50_ = 4.2 ± 1.0 µM	Kim et al., 2014
*Ecklonia cava* (Laminariaceae/whole)	Dieckol (Ethanol extract)	*In vitro* SARS-CoV- 3CL(pro)	FRET	0–200 µM	Positive controls: hesperetin (60 μM), daidzein (105 μM), aloeemodin (132 μM)	Inhibition of the SARS-CoV- 3CL(pro) activity (IC_50_ = 2.7 ± 0.6 µM)	Park et al., 2013
*Paulownia tomentosa* (Thunb.) Steud. (Scrophulariaceae/fruits)	Tomentin E (Methanol extract)	*In vitro* SARS-CoV- PLpro	Fluorogenic assay	0, 6.25, 12.5, 25 µM	NS	A dose-dependent inhibition of SARS-CoV-PLpro (IC_50_ = 5.0 ± 0.06 µM)	Cho et al., 2013
*Quercus infectoria* G. Olivier (Fagaceae/bark)	Ethanol-water extract	*In vitro*	Reverse phase high performance liquid chromatography (RP-HPLC)	330 µg/ml	Methanol solution (negative control)	Inhibition of ACE activity by 93.9 ± 2.5%	Sharifi et al., 2013
*Rheum* sp. *Polygonum* sp. (Polygonaceae/whole)	Emodin	*In vitro* Vero cells	Luciferase assay	0, 10, 50, 100, 200, and 400 µM	NS	Blockage of the binding SARS-CoV S protein and ACE2 Minimal active concentration: 10 µM IC_50_ = 200 µM	Ho et al., 2007
*Salvia miltiorrhiza* Bunge (Lamiaceae/roots)	Cryptotanshinone (n-hexane extract)	*In vitro* SARS-CoV- PLpro	FRET	0–200 µM/30 min	NS	A dose- and time-dependent inhibition of the SARS-CoV- PLpro activity in a slow-binding inhibition mechanism (IC_50_ = 0.8 ± 0.2 µM)	Park et al., 2012a
	Dihydrotanshinone I (n-hexane extract)	*In vitro* SARS-CoV- 3CL(pro)	FRET	0–200 µM/60 min	NS	A dose-dependent but not time-dependent inhibition of the SARS-CoV- 3CL(pro) activity (IC_50_ = 14.4 ± 0.7 µM)	
*Sambucus javanica* subsp. chinensis (Lindl.) Fukuoka (Adoxaceae/stem)	95% ethanol extract	*In vitro* HCoV-NL63 in LLC-MK2 cells	Cytopathic effect inhibition	0, 1, 10, and 50 μg/ml/36 h	Virus-infection only with no test extract	Inhibition of: -Viral cytopathicity (IC_50_ = 1.17 ± 0.75 μg/ml) -Virus attachment (IC_50_ = 15.75 ± 6.65 μg/mL) -Plaque formation 4.67 ± 1.21 μg/mL	Weng et al., 2019
	Caffeic acid			0, 10, 50, and 100 μM/36 h		Inhibition of viral cytopathicity (IC_50_ = 3.54 ± 0.77 μM)	
	Chlorogenic acid					Inhibition of viral cytopathicity (IC_50_ = 43.45 ± 6.01 μM)	
	Gallic acid					Inhibition of viral cytopathicity (IC_50_ = 71.48 ± 18.40 μM)	
*Scutellaria baicalensis *Georgi (Labiatae/whole)	Scutellarein	*In vitro*	FRET	0.01–10 µM	Inhibition of the SARS-CoV helicase by affecting the ATPase activity	Inhibition of the SARS-CoV helicase by affecting the ATPase activity (IC_50_ = 0.86 ± 0.48 µM)	Yu et al., 2012
*Torreya nucifera* (L.) Siebold & Zucc. (Taxaceae/leaves)	Amentoflavone (Ethanol extract)	*In vitro* SARS-CoV- 3CL(pro)	FRET	0–300 µM	Positive controls: Apigenin (IC_50_ = 280.8 ± 21.4 µM) Luteolin (IC_50_ = 20.0 ± 2.2 µM) Quercetin (IC_50_ = 23.8 ± 1.9 µM)	A dose-dependent inhibition of SARS-CoV- 3CL(pro) activity (IC_50_ = 8.3 ± 1.2 µM)	Ryu et al., 2010
*Tribulus terrestris* L. (Zygophyllaceae/fruits)	Terrestrimine (Methanol extract)	*In vitro* SARS-CoV PLpro	Fluorogenic assay	1, 10, 100, 1,000 µM	NS	Inhibition of SARS-CoV - PLpro with IC_50_ = 15.8 ± 0.6 µM	Song et al., 2014
———————	Lianhuaqingwen (Herbal mixture) dissolved in DMSO and then in serum-free DMEM	*In vitro* SARS-CoV-2 virus propagated in Vero E6 cells	Cytopathic effect inhibition	0–600 µg/ml/72 h	Remdesivir (5 µM)	Inhibition of the SARS-CoV-2 replication (IC_50_ = 412.2 µg/ml *vs* 0.651 µM for the control) Inhibition of the plaque formation of the SARS-CoV-2 Reduction of TNF-α, IL-6, CCL-2/MCP-1, and CXCL-10/IP-10 expression	Runfeng et al., 2020
*——————–*	Herbacetin	*In vitro* SARS-CoV- 3CL(pro)	FRET	1, 2.5, 20 µM/16 h	NS	A dose-dependent inhibition of SARS-CoV- 3CL(pro) activity (IC_50_ = 33.17 µM)	Jo et al., 2020
*——————–*	Pectolinarin	*In vitro* SARS-CoV- 3CL(pro)	FRET	1, 2.5, 20 µM/16 h	NS	A dose-dependent inhibition of SARS-CoV- 3CL(pro) activity (IC_50_ = 37.78 µM)	Jo et al., 2020
*——————–*	Rhoifolin	*In vitro* SARS-CoV- 3CL(pro)	FRET	1, 2.5, 20 µM/16 h	NS	A dose-dependent inhibition of SARS-CoV- 3CL(pro) activity (IC_50_ = 27.45 µM)	Jo et al., 2020

FRET, Fluorescence resonance energy transfer; GSEA, Microarray and Gene Set Enrichment Analysis; NS, Not specified.

## SARS-CoV-2

SARS-CoV-2 belongs to the *β* genus, Nidovirales order of the Coronaviridae family. SARS-CoV-2 is an enveloped, single (+) stranded RNA, with symmetric helical nucleocapsid (Khan et al., 2020). The virus encodes twenty different proteins including four main structural proteins (S: spike; E: envelope; M: membrane; N: nucleocapsid), and several nonstructural proteins such as RNA-dependent RNA polymerase (RdRp), coronavirus main protease (3CLpro), and papain-like protease (PLpro) (Chen et al., 2020).

The angiotensin converting enzyme II (ACE2) was found to be a key functional receptor for the SARS-CoV-2 allowing its attachment to human and bat cells and therefore its replication (Walls et al., 2020; Zhou et al., 2020). SARS-CoV-2 binds the host cells through interaction between the receptor binding motif of the spike protein—receptor binding domain (RBD) and the ACE2 receptor. This interaction will trigger conformational changes of the C-terminal S2 subunit (responsible for virus-cell membrane fusion) of the spike protein. The complex S protein-ACE2 is then proteolytically processed by the host cell-type 2II transmembrane serine protease TMPRSS2 leading to the ACE2 cleavage and therefore to viral entry into the host cell (Jiang et al., 2020; Rabi et al., 2020). After entry and uncoating, the genomic RNA is translated into two polyproteins (pp1a and pp1ab) which undergo a proteolytic cleavage generating 15–16 nonstructural proteins. The double-membrane vesicle is then formed from the rearrangement of cellular membrane induced by the nonstructural proteins. On the other hand, the genomic RNA is transcribed into subgenomic RNA which in turn leads to the synthesis of structural (spike, envelope, membrane, and nucleocapsid) and accessory proteins. Finally, virions are assembled in the ER-Golgi intermediate complex, and then released *via* the secretory pathway (Fung and Liu, 2020).

SARS-CoV-2 shares several genetic and clinical similarities with other coronaviruses of the *β* genus such as SARs-CoV and NL63 (Fani et al., 2020). Indeed, the entry of both viruses needs their interaction with the ACE2 receptor. However, some differences have been reported among these strains such as the length of the S protein and the structure of the receptor binding region (Ceccarelli et al., 2020). On the other hand, a high nucleotides homology has been found between SARS-CoV-2 and SARS-CoV in addition to a high homology (95–100%) that has been demonstrated between the proteins of the two strains. Actually, S2 and N proteins of SARS-CoV-2 and SARS-CoV share 99 and 90% similarities, respectively (Xu J. et al., 2020).

## Natural Products With Anti-SARS-CoV-2 Effects

Runfeng et al. (2020) studied the inhibitory effects and anti-inflammatory potential of a Chinese herbal mixture called Lianhuaqingwen (a mixture of 11 medicinal species, a mineral medicine called gypsum and menthol) against SARS-CoV-2 (**Table 2**). Traditionally, Lianhuaqingwen has been widely used to treat fever, cough, fatigue, influenza, bronchitis, pneumonia, and early stage of measles (Ding et al., 2017), and has been included in phase II clinical trial in the USA (Gao et al., 2020). This herbal mixture was recommended by the Chinese National Health Commission to treat or manage COVID-19 (Yang Y. et al., 2020). The anti-SARS-CoV-2 activity was assessed in Vero E6 cells using cytopathic effect inhibition and plaque reduction assays. The herbal mixture inhibited SARS-CoV-2 replication in a dose-dependent manner with an IC_50_ of 411.2 µg/ml. Furthermore, the mixture was able to suppress the release of pro-inflammatory cytokines (TNF-α, IL-6, CCL-2/MCP-1, and CXCL-10/IP-10) in a dose-dependent manner (Runfeng et al., 2020). These results could be interesting since the cytokine storm has been shown to be one of the COVID-19 lethal complications. In a previous study, among the 61 molecules identified in this herbal mixture, seven (arctiin, forsythoside A, gallic acid, isoliquiritigenin, kaempferol, rutin, and secoxyloganin) exhibited important antiviral activities with IC_50_ ranging from 4.9 ± 0.1 (kaempferol) to 47.8 ± 1.5 µM (secoxyloganin) (Wang et al., 2016). Wang et al. (2020) reported results in four COVID-19 patients treated using a combination of lopinavir/ritonavir (Kaletra^®^) and arbidol with capsules of Shufeng Jiedu (a Chinese traditional medicine). After treatment, three patients were found COVID-19 negative and experienced significant improvements of the symptoms. In another study on 132 patients with COVID-19 living in northeast Chongqing (China), the traditional Chinese medicine was applied in almost 92% of them. The study concluded that the best therapeutic approach was a combination of Kaletra and the traditional medicine (Wan et al., 2020). Recently, Lung et al. (2020) demonstrated that theaflavin could be used as an important anti-SARS-CoV-2 drug using *in silico* approaches. Indeed, theaflavin showed promising docking affinities in the catalytic pocket of the SARS-CoV-2 RNA-dependent RNA polymerase. Nevertheless, it is worthy to point out that their bioavailability could limit their use since they are not absorbed in relevant amounts and that the theaflavin skeleton was found to resist to the degradation by the microbiota (Pereira-Caro et al., 2017). By searching single cohort studies undertaken regarding the efficacy of herbal medicines against SARS and H1N1 influenza viruses, it has been concluded that medicinal species, usually used as herbal formula, could be an interesting preventive approach for high-risk populations (medical staff and their families’ members, people living in COVID-19 outbreak areas, old populations). Six herbal species were found to be the most frequently used including *Astragalus mongholicus *Bunge, *Glycyrrhiza glabra* L., *Saposhnikovia divaricata (*Turcz. ex Ledeb.) Schischk., *Atractylodes lancea* (Thunb.) DC., *Atractylodes macrocephala* Koidz., *Lonicera japonica* Thunb., and *Forsythia suspensa* (Thunb.) Vahl. These species are the ingredients of the Chinese traditional medicine Yupingfeng powder (Luo et al., 2020). On the other hand, the ethanol extract of *Sambucus javanica* subsp. chinensis (Lindl.) Fukuoka stem exerted promising anti-human coronavirus NL63 effects with IC_50_ ranging from 1.17 (virus yield) to 15.75 µg/ml (virus attachment). The extract significantly decreased virus yield, plaque formation, and virus attachment. Furthermore, three of its major phenolic acids (caffeic, chlorogenic, and gallic acid) were shown to inhibit the NL63 replication and virus attachment. Caffeic acid was the most potent phenolic acid (Weng et al., 2019). Phenolic acids are characterized by their metabolizing ability by the microbiota enhancing their bioavailability. Moreover, their antiviral potential could be increased with the alkyl chain length (Kumar and Goel, 2019). However, their efficacy is still controversial due to their low absorption and instability in alkaline and neutral media, which could limit their use in pure form. Therefore, the clinical utility of phenolic compounds as anti-SARS-CoV-2 agents remains debatable since their bioavailability, delivery mechanisms and efficient doses should be further studied using *in vivo* models.

**Table 2:** Traditional uses of the medicinal species and mixtures with possible anti-SARS-CoV-2 effects.

Plant (family)	Traditional uses	References
**Medicinal species**		
*Alnus japonica (Thunb.) Steud.* (Betulaceae)	Cancer, Blood and lymphatic disorders, Gastroenteric disorders, Fever.	Chi et al., 2018
*Angelica keiskei* (Miq.) Koidz. (Umbelliferae)	Tonic, Galactagogue, Diuretic, Laxative, Analeptic.	Kil et al., 2017 Du et al., 2019
*Berberis integerrima* Bunge *(*Berberidaceae*)*	Hypertension, Abdominal ache, Blood purification, Fever.	Nasab and Khosravi, 2014 Sadat-Hosseini et al., 2017
*Cibotium barometz* (L.) J. Sm. (Dicksoniaceae)	Osteoporosis, Osteoarthritis, Inflammations, Rheumatism, Lumbago, Dysuria, Age-related leucorrhoea.	Shi et al., 2020 Wu and Yang, 2009
*Crataegus laevigata* (Poir.) DC. (Rosaceae)	Hyperlipidemia, Arteriosclerosis, Hypertension, Cardiac disorders.	Čopra-Janićijević et al., 2018
*Ecklonia cava* (Lessoniaceae)	Inflammations, Asthma, Diabetes, Cancer.	Cho et al., 2020 Yun et al., 2018 Park E. Y. et al., 2012
*Gentiana scabra* Bunge (Gentianaceae)	Inflammatory skin diseases, Gallbladder disorders, Jaundice, Leucorrhea.	Yang et al., 2019
*Onopordum acanthium* L. (Asteraceae)	Hypertension, Homeostasis, Bacterial infections.	Sharifi et al., 2013
*Paulownia tomentosa* (Thunb.) Steud. (Scrophulariaceae)	Bacterial and viral infections, Inflammations, Asthma, Hypertension, Hemorrhoids, Gonorrhea, Erysipelas.	Ji et al., 2015 Lee et al., 2018
*Cullen corylifolium* (L.) Medik. (Leguminosae)	Eczema, Osteoporosis, Colitis, Pollakiuria, Leukoderma, Asthma, Spermatorrhea, Bleeding.	Xu et al., 2020a Zhu et al., 2019
*Quercus infectoria* G. Olivier (Fagaceae)	Diarrhea, Dysentery, Gonorrhea, Hemorrhages, Infections, Inflammations.	Tayel et al., 2018 Chokpaisarn et al. (2017)
*Salvia miltiorrhiza* Bunge (Lamiaceae)	Inflammations Cardiovascular and circulatory disorders, Menstrual disorders, Atherosclerosis, Cancer, Hyperglycemia.	Wei et al., 2020 Liang et al., 2020
*Sambucus javanica* subsp. chinensis (Lindl.) Fukuoka (Adoxaceae)	Bacterial infections, Inflammations, Liver disorders.	Zhang et al., 2010
Senna tora (L.) Roxb. (Fabaceae)	Constipation, Liver disorders, Inflammations, Vision disorders.	Lee et al., 2019
*Taxillus chinensis* (DC.) Danser (Loranthaceae)	Liver and kidney disorders, Miscarriage, Rheumatic arthralgia, Metrorrhagia, Pregnancy-related Bleeding, Dizziness.	Liu et al., 2019
*Torreya nucifera* (L.) Siebold & Zucc. (Taxaceae)	Stomachache, Hemorrhoids, Rheumatoid arthritis, Nervous disorders, Viral infections.	Kalpana et al., 2019
*Tribulus terrestris* L. (Zygophyllaceae)	Inflammation, Oxidative stress, Cancer, Cardiovascular disorders, Hormonal disorders, Muscle aches, Chest pain, Pruritus.	Tian et al., 2020
*Dioscorea polystachya* Turcz. (Dioscoreaceae)	Inflammations, Liver disorders, Diabetes, Hyperthyroidism, Digestive disorders, Cancer.	Koo et al., 2017 Ma et al., 2018
**Herbal mixtures**		
**Lianhuaqingwen** **Composition** Forsythiae Fructus [*Forsythia suspensa* (Thunb.) Vahl], Lonicerae Japonicae Flos [*Lonicera japonica* Thunb.], Ephedrae Herba (honey-fried) [*Ephedra sinica Stapf*, *Ephedra intermedia* Schrenk & C. A. Mey. or *Ephedra equisetina* Bunge.], Armeniacae Semen Amarum (stir-baked) [*Prunus armeniaca* L. var. *ansu* Maxim., *Prunus sibirica* L., *Prunus mandshurica* (Maxim.) Koehne or *Prunus armeniaca* L.], Gypsum Fibrosum, Isatidis Radix [*Isatis tinctoria* L.], Dryopteris Crassirhizomatis Rhizoma [*Dryopteris crassirhizoma* Nakai], Houttuyniae Herba [*Houttuynia cordata* Thunb.], Pogostemonis Herba [*Pogostemon cablin* (Blanco) Benth], Rhei Radix & Rhizoma [*Rheum palmatum* L., *Rheum tanguticum* (Maxim. ex Regel) Balf. or *Rheum officinale* Baill.], Rhodiolae Crenulatae Radix et Rhizoma [*Rhodiola crenulata* (Hook.f. & Thomson) H.Ohba], menthol and Glycyrrhizae Radix et Rhizoma [*Glycyrrhiza uralensis* Fisch. ex DC., *Glycyrrhiza inflata* Batalin, or *Glycyrrhiza glabra* L.].	Respiratory tract infectious diseases, Viral infections, Inflammations, Fever.	Runfeng et al., 2020 Ding et al., 2017 Gao et al., 2020
**Shu Feng Jie Du** **Composition** Rhizoma Polygoni Cuspidati [*Reynoutria japonica* Houtt.] Fructus Forsythiae [*Forsythia suspensa *(Thunb.) Vahl] Radix Isatidis [*Isatis tinctoria* L.] Radix Bupleuri [*Bupleurum chinense* DC.] Herba Patriniae [*Patrinia scabiosifolia* Link] Herba Verbenae [*Verbena officinalis* L.] Rhizoma Phragmitis [*Phragmites australis* subsp. australis] Radix Glycyrrhizae [*Glycyrrhiza uralensis* Fisch. ex DC.]	Influenza, Viral infections, Immune regulation.	Song et al., 2013 Yang Y. et al. (2020) Xia et al., 2020

Wen et al. (2011) evaluated 200 Chinese herbal extracts for their anti-SARS-CoV effect using a cell-based assay. Among them, six extracts [rhizomes of *Gentiana scabra* Bunge; tuber of *Dioscorea polystachya* Turcz.; seed of *Senna tora* (L.) Roxb.; stem and leaves of *Taxillus chinensis* (DC.) Danser; and two extracts of *Cibotium barometz* (L.) J.Sm. rhizome] were found to significantly inhibit SARS-CoV growth and replication. IC_50_ values ranged from 25 to 200 μg/ml. By using FRET assay, the study demonstrated that extracts obtained from tuber of *Dioscorea polystachya* Turcz. and rhizome of *Cibotium barometz* (L.) J.Sm. caused marked inhibition of SARS-CoV- 3CL protease with IC_50_ of 39 and 44 μg/ml, respectively.

**Figure 1:**
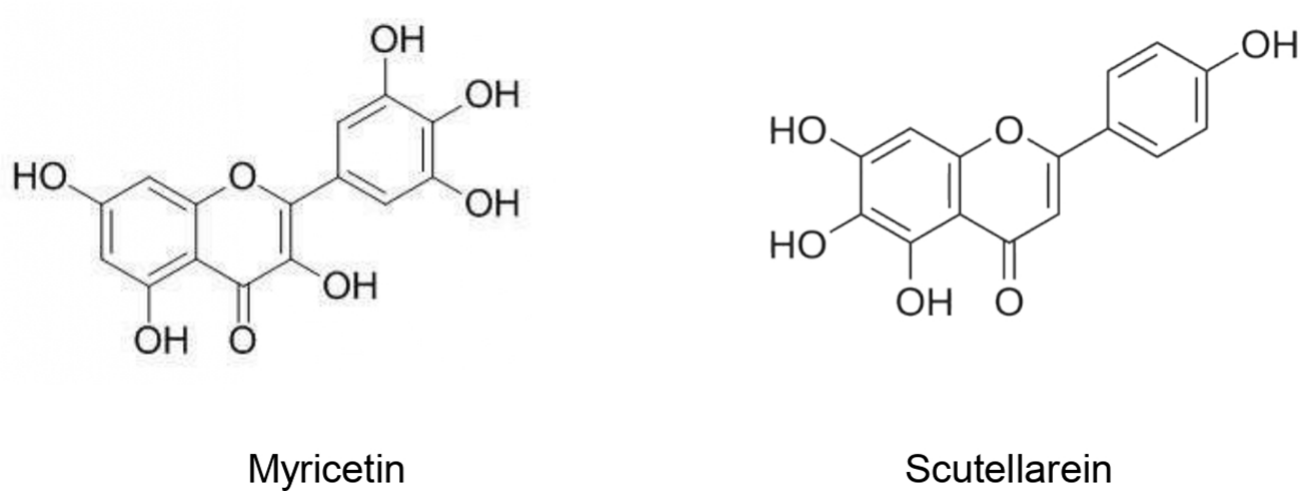
Natural products that act as SARS-CoV helicase activity.

Owing to its importance as a key protein for SARS-CoV genome replication, SARS-CoV helicase still remains a target of novel antiviral drugs. Sixty-four natural molecules originated from 15 medicinal species were evaluated regarding their inhibitory activity of SARS-CoV helicase. Myricetin and scutellarein (**Figure 1**) significantly inhibited the SARS-CoV helicase activity. At 10 µM, myricetin (IC_50_ = 2.71 ± 0.19 µM) and scutellarein (IC_50_ = 0.86 ± 0.48 µM) were able to inhibit 90% of the ATPase activity of the SARS-CoV helicase. Accordingly, Myricetin and scutellarein were suggested to be promising future anti-SARS drugs (Yu et al., 2012).

SARS-CoV-2 life-cycle related proteins are considered promising targets of antiviral drugs. Therefore, molecules and/or products able to inhibit these proteins may be used to treat or even prevent the SARS-CoV-2 infections (**Table 1**).

## Natural Products as ACE2-Blockers

The penetration of the SARS-CoV-2 genome into the host cells occurs as a result of the SARS-CoV-2 spike protein binding to host receptors (Sigrist et al., 2020). By using phylogenetic analysis and critical site of ACE2 structure, different animals such as cat, pigeon, and sheep were predicted to be important intermediate hosts for SARS-CoV-2 (Qiu et al., 2020). Hoffmann et al. (2020) demonstrated that the ACE2 receptor is used by SARS-CoV-2 to enter human cells. Moreover, they reported that the use of TMPRSS2 inhibitors may be a promising therapeutic approach against SARS-CoV-2. TMPRSS2 is a transmembrane serine protease that cleaves both ACE2 and the S protein. Recently, Ortega et al. (2020) used *in silico* approaches to understanding the relationship between changes in SARS-CoV-2 Spike protein and ACE2 receptor. They demonstrated superior affinity of SARS-CoV-2 spike protein towards human ACE2 as compared to that of the Bat-CoV spike and ACE2. This study supported the idea that the ACE2 receptor may be the key “bridge” used by SARS-CoV-2 to transmit among humans. Chen et al. (2020) confirmed that although SARS-CoV and SARS-CoV-2 RBD of spike glycoprotein had 72% of structural similarities, SARS-CoV-2 RBD exhibited higher interaction with ACE2. ACE2 inhibitors are thought to indirectly alter the RBD binding site and therefore block SARS-CoV-2 infection. Likewise, Wrapp et al. (2020) found that the SARS-CoV-2 spike exhibited a higher affinity to ACE2 than SARS-CoV. Adedeji et al. (2013) demonstrated that early blocking of SARS-CoV with ACE2 inhibitors was one of the mechanisms used by novel efficient anti-SARS drugs. Nonetheless, it has been shown in three recent studies on COVID-19 that hypertension and diabetes mellitus significantly enhanced the risk of COVID-19 infection, in spite of using ACE2 inhibitors (Guan et al., 2019; Yang X. et al., 2020; Zhang J. J. et al., 2020). ACE2 inhibitors, angiotensin II type-I receptor blockers, and ibuprofen lead to ACE2 upregulation which justifies the urgent need to use and/or identify alternative ACE2 blockers (Fang et al., 2020). Hence, medicinal plants-derived products or natural products able to selectively block the ACE2 receptor without inhibiting the enzyme activity may be useful to prevent and/or treat SARS-CoV-2 spread in humans without increasing ACE2 expression in patients and therefore increased risk for COVID-19.

**Figure 2:**
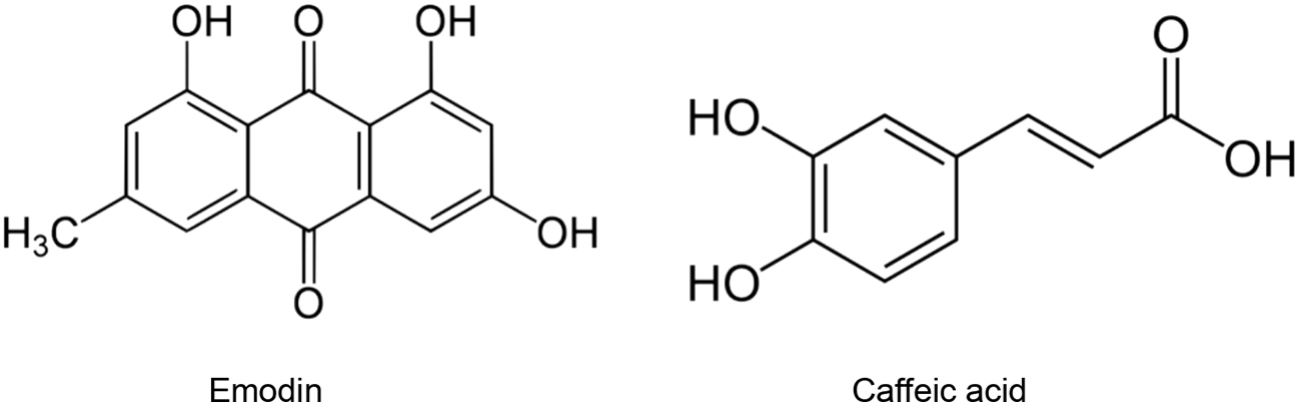
Natural products that act as inhibitors of ACE2.

Since important similarities exist between the sequences of ACE and ACE2 (Guy et al., 2005), molecules with inhibitory effects toward ACE may exert the same effect toward ACE2 and thus lead to reduce the viral entry. However, further studies should be undertaken to evaluate this hypothesis. Patten et al. (2016) reviewed the medicinal plants for their inhibitory effects on ACE2. They reported 141 medicinal species belonging to 73 families and 49 purified natural compounds with documented ACE inhibitory potential. Moreover, 16 medicinal species (16 families) were found to be able to block the angiotensin type 1A receptor *in vitro*. Sharifi et al. (2013) identified four Iranian medicinal species able to inhibit more than 80% of ACE activity *in vitro*. These active species were: *Berberis integerrima* Bunge., *Crataegus laevigata* (Poir.) DC., *Onopordum acanthium* L., and *Quercus infectoria* G. Olivier. At 330 µg/ml, Q*uercus infectoria* G. Olivier. was found to be the most active and caused 94% ACE inhibition. This important inhibitory activity might be attributed to its higher phenolic content and enhanced antioxidant potential. In spite of the important ACE inhibition and antioxidant activities exhibited by *Q. Infectoria* extract, the presence of condensed tannins compromised its usefulness due to their interference in the functions of ACE. On the other hand, *B. integerrima, C. microphylla* and *O. acanthium* were found to have both important ACE inhibitory activities and enhanced antioxidant potential without the presence of tannins (Sharifi et al., 2013 *)*. These species could be promising sources of antiviral molecules. Indeed, viral infections are accompanied by oxidative stress which in turn promotes virus replication. Antioxidant species decrease the ROS production in infected cells and target different oxidative stress-related signalling pathways resulting in reduction of viral spread (Fedoreyev et al., 2018). Walls et al. (2020) demonstrated that SARS-CoV-2 and SARS-CoV bind with similar affinities to ACE2. In another study, 25 Chinese herbal families were found to significantly inhibit the interaction SARS-CoV – ACE2. Among them, species belonging to Polygonaceae, Labiatae, Oleaceae, Magnoliaceae, Lauraceae, and Nelumbonaceae exhibited the most important inhibitory effects. These inhibitory effects were attributed to emodin (1,3,8-trihydroxy-6-methylanthraquinone) (**Figure 2**) produced in high levels in in genus Rheum and Polygonum. Emodin blocked the interaction SARS-CoV S protein and ACE2 in a dose-dependent manner with an IC_50_ of 200 µM (Ho et al., 2007).

## Natural Products Targeting the TMPRSS2

**Figure 3:**
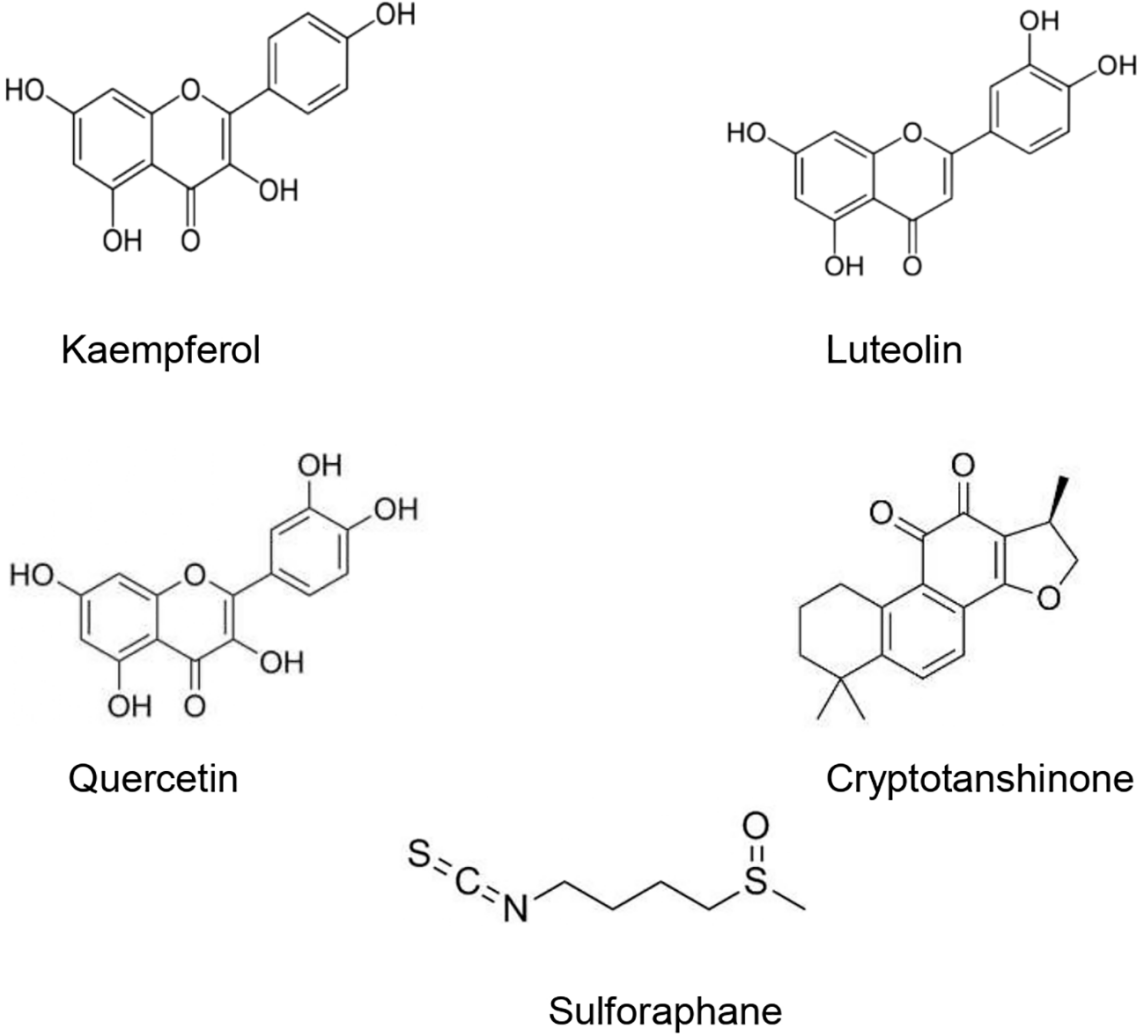
Natural products that act as inhibitors of TMPRSS2.

The type II transmembrane serine-proteinase serine type 2 RSS2 type II transmembrane serine protease that cleaves the S spike proteins of SARS-CoV and MERS and ACE2 (Iwata-Yoshikawa et al., 2019). Recently, Hoffmann et al. (2020) demonstrated that besides using ACE2 receptor to enter the host cells, SARS-CoV-2 uses also TMPRSS2 for S protein priming. After the interaction between the S spike protein (SARS-CoV-2) and the ACE2 (host cell), the complex is cleaved by the TMPRSS2 to facilitate viral entry (Rabi et al., 2020). Matsuyama et al. (2020) found that an important TMPRSS2 expression in cells makes them highly susceptible to SARS-CoV-2. Since SARS-CoV-2 viral entry is conditioned by its binding to the ACE2 receptor, and the latter should be cleaved by the TMPRSS, finding agents able to suppress or downregulate the TMPRSS2 expression in human cells could represent a promising therapeutic or preventive approach (Schlagenhauf et al., 2020). Several studies have demonstrated that natural products could downregulate or suppress TMPRSS2. It has been shown that kaempferol was able to inactivate TMPRSS2 expression by 49.14 and 79.48% at 5 and 15 μM, respectively (Da et al., 2019). Likewise, sulforaphane (an isothiocyanate) was found to downregulate the TMPRSS2 expression through the release and translocation of the Nrf2 (nuclear factor (erythroid-derived 2)-like 2) (Gibbs et al., 2009; Meyer and Jaspers, 2015). Mamouni et al. (2018) found that a standardized flavonoids formulation including luteolin, quercetin, and kaempferol significantly suppressed TMPRSS2 expression. In spite of the diverse biological effects attributed to the three flavonoids, this study has demonstrated that at low concentrations, they exhibited an important synergic effect. Nonetheless, the efficacy and safety of these compounds in COVID-19 patients is still unclear. Moreover, modes of administration, the health of the patients’ digestive system, and disease stage may limit the clinical usefulness of such formulations and compounds (Fuzimoto and Isidoro, 2020). Xu et al. (2012) demonstrated that cryptotanshinone at 0.5 µM effectively exhibited anti-TMPRSS2 activity (**Figure 3**).

## Natural Products Targeting the Papain-Like Proteinase (Plpro)

PLpro is one of the nonstructural proteins encoded by the SARS-CoV-2 genome. This protease is vital for virus replication since it contributes to the cleavage of viral polyproteins (PP1A and PP1AB) into effector proteins (Jiang et al., 2020). Moreover, PLpro has been found to be an antagonist of the host’s innate immunity (Yuan et al., 2015; Li et al., 2016). Actually, PLpro was shown to target the interferon production by blocking the IRF3 phosphorylation, dimerization, and nuclear translocation and NF-κB signaling pathways (by preventing IκBα degradation) (Wong et al., 2016). These effects were shown to occur in Toll-like receptor 3 and retinoic acid-inducible gene 1 pathways. Moreover, it has been demonstrated that SARS-CoV PLpro inhibits the TLR7 pathway *via* inactivation of TRAF3/6-TBK1-IRF3/NF-κB/AP1 signalling pathways (Yuan et al., 2015).

Recently, Arya et al. (2020) screened FDA-approved drugs for their *in silico* inhibitory potential of PLpro. They demonstrated that sixteen FDA-approved drugs (Biltricide, Cinacalcet, Procainamide, Terbinafine, Pethidine, Labetalol, Tetrahydrozoline, Ticlopidine, Ethoheptazine, Levamisole, Amitriptyline, Naphazoline, Formoterol, Benzylpenicillin, Chloroquine, and Chlorothiazide) exhibited important binding affinity to SARS-CoV-2 PLpro suggesting their possible effectiveness as anti-SARS-CoV-2 agents. Likewise, Disulfiram (an alcohol-aversive drug) was found to be a competitive inhibitor of SARS-CoV PLpro (Lin et al., 2018).

**Figure 4:**
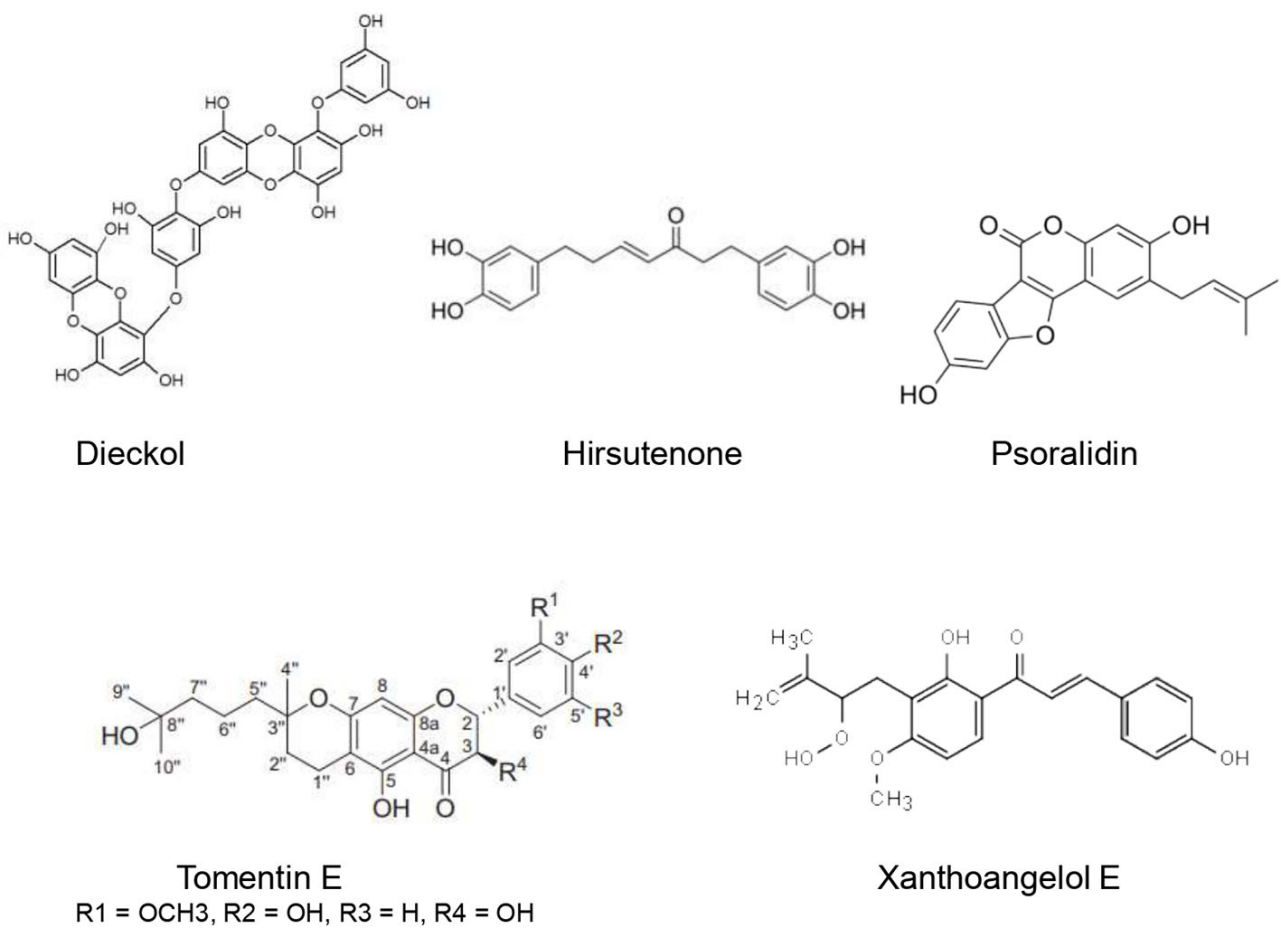
Natural products that act as inhibitors of PLpro.

Several compounds have been shown to target the SARS-CoV PLpro (**Figure 4**).

### Cinnamic Amides From *Tribulus terrestris*

Several natural compounds were found to possess promising PLpro inhibitory effects. Indeed, Song et al. (2014) demonstrated that six cinnamic amides (N-trans-Feruloyloctopamine, N-trans-Coumaroyltyramine, N-trans-Caffeoyltryamine, Terrestrimine, N-trans-Feruloyltryamine, and Terrestriamide) extracted from *Tribulus terrestris* L. fruits were able to inhibit SARS-CoV PLpro in a dose-dependent manner. PLpro inhibitory IC_50_ of these compounds were found to be 15.8–70.1 µM. Terrestrimine [(E)-N-(1-hydroxy-2-(4-hydroxyphenyl)-2- oxoethyl)-3-(4-hydroxy-3-methoxypheny) acrylamide] showed the best inhibitory activity of SARS-CoV PLpro with an IC_50_ of 15.8 ± 0.6 µM. The presence of a polar substituent (ketone or alcohol) on the methylene groups (C8’and C7’) was associated with enhanced inhibitory activity.

### Flavonoids From *Cullen corylifolium* (L.) Medik.

Ethanolic extract of *Cullen corylifolium* (L.) Medik. seeds showed an important inhibitory effect of SARS-CoV PLpro with an IC_50_ of 15 µg/ml. Furthermore, six flavonoids present in the extract (Bavachinin, neobavaisoflavone, isobavachalcone,40 –O-methylbavachalcone, psoralidin, and corylifol A) inhibited SARS-CoV PLpro activity in a dose-dependent manner with IC_50_ estimated to be 4.2–38.4 µM. The highest inhibitory effect was exerted by psoralidin (IC_50_ = 4.2 ± 1.0 µM) and isobavachalcone (IC_50_ = 7.3 ± 0.8 µM) (Kim et al., 2014).

### Flavonoids From *Paulownia tomentosa* (Thunb.) Steud.

Cho et al. (2013) identified five new geranylated flavonones, tomentin A, tomentin B, tomentin C, tomentin D, tomentin E from the ethanolic extract of *Paulownia tomentosa* (Thunb.) Steud. fruits. These flavonoids besides seven other knowns resulted in significant inhibition of SARS-CoV PLpro in a dose-dependent manner with IC_50_ of 5.0 and 14.4 μM. Tomentin E exhibited the highest inhibitory effect with an IC_50_ of 5.0 ± 0.06 µM. It has been found that molecules with 3,4-dihydro-2H-pyran moiety possessed higher inhibition. The *P. tomentosa* flavonoids were found to be reversible, mixed inhibitors.

### Chalcones From *Angelica keiskei* (Miq.) Koidz

Park et al. (2016) investigated the inhibitory potential of nine alkylated chalcones (isobavachalcone, 4-hydroxyderricin, xanthoangelol, xanthoangelol F, xanthoangelol D, xanthoangelol E, xanthoangelol B, xanthoangelol G, xanthokeistal A) and four coumarins extracted from the ethanolic extract of *Angelica keiskei* (Miq.) Koidz. The alkylated chalcones inhibited SARS-CoV PLpro in a significant dose-dependent manner with IC_50_ ranging from 1.2 ± 0.4 to 46.4 ± 7.8 µM. On the other hand, the analyzed coumarins did not show a significant inhibitory effect toward SARS-CoV PLpro. Kinetic studies revealed that isobavachalcone was a mixed inhibitor whereas the other chalcones were noncompetitive. Interestingly, xanthoangelol E, an –OOH substituted analogue, exhibited the most enhanced inhibitory effect (IC_50_ = 1.2 ± 0.4 µM), which was 40-fold higher when compared to other tested chalcones. This important inhibitory activity of xanthoangelol E was confirmed using *in silico studies.*

### Tanshinones From *Salvia miltiorrhiza* Bunge

*Salvia miltiorrhiza* Bunge ethanolic extract (30 µg/ml) caused 88% inhibition of SARS-CoV PLpro. Moreover, seven bioactive tanshinones (tanshinone IIA, tanshinone IIB, methyl tanshinonate, cryptotanshinone, tanshinone I, dihydrotanshinone I, and rosmariquinone) were identified from the n-hexane fraction. These tanshinones were evaluated with regard to inhibition of SARS-CoV PLpro activity using a fluorometric assay. Both molecules exhibited important inhibitory time-dependent activities with IC_50_ of 0.8 to 30 µM. The dimethyl tetrahydronaphthalen structure was associated with enhanced inhibitory potential. The results obtained showed that cryptotanshinone was the most potent inhibitor of SARS-CoV PLpro with an IC_50_ of 0.8 ± 0.2 µM. Kinetic investigations demonstrated that rosmariquinone was a mixed-type inhibitor of SARS-CoV PLpro, whereas the other tanshinones were noncompetitive inhibitors (Park et al., 2012a).

### Diarylheptanoids From *Alnus japonica* (Thunb.) Steud.

Park et al. (2012b) used activity-guided fractionation to identify nine diarylheptanoids (platyphyllenone, hirsutenone, platyphyllone, platyphyllonol-5-xylopyranoside, hirsutanonol, oregonin, rubranol, rubranoside B, and rubranoside A) from the ethanol extract of *Alnus japonica (Thunb.) Steud.* They evaluated their SARS-CoV PLpro inhibitory effect using a continuous fluorometric assay. The results showed that hirsutenone, hirsutanonol, oregonin, rubranol, rubranoside B, and rubranoside A exerted a significant dose-dependent inhibitory activity towards SARS-CoV PLpro with IC_50_ ranging from 3 to 44.5 µM. Hirsutenone possessed the most potent inhibitory effect with IC_50_ of 4.1 ± 0.3 µM which was less important than that of the reference inhibitor curcumin (5.7 µM). The enhanced inhibitory potential of diarylheptanoids seems to be related to the presence of α,β-unsaturated carbonyl, and catechol groups.

## Natural Products Targeting the Chymotrypsin-Like Protease [3CL(pro)]

**Figure 5:**
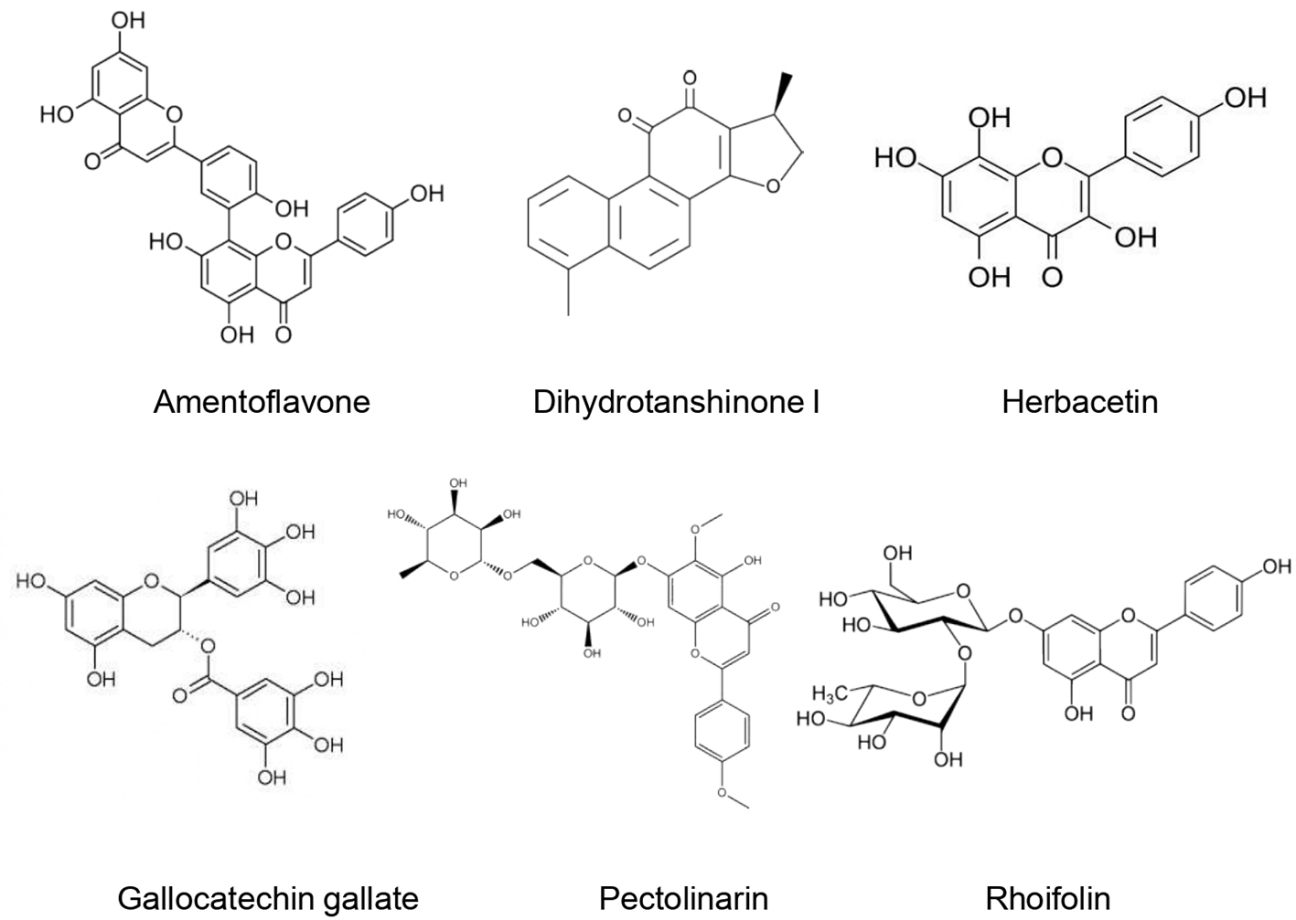
Natural products that act as inhibitors of 3CL(pro).

3CL(pro) belongs to the 16 nonstructural proteins of the SARS-CoV-2. Since it plays an important role in the SARS-CoV-2 replication process polyproteins, 3CL(pro) is considered a potential therapeutic target for anti-COVID-19 drugs (Zhang L. et al., 2020). Different natural compounds exhibited promising anti3CL(pro) activities (**Figure 5**).

### Alkylated Chalcones From *Angelica keiskei* (Miq.) Koidz

Inhibitory potential toward SARS-CoV- 3CL(pro) of alkylated chalcones and coumarins extracted from *Angelica keiskei* (Miq.) Koidz was investigated using a fluorescence resonance energy transfer (FRET) method. Except for coumarins, alkylated chalcones exhibited marked inhibitory effects in a dose-dependent fashion. IC_50_ ranged from 11.4 ± 1.4 to 129.8 ± 10.3 µM. Moreover, xanthoangelol E (**Figure 5**) was found to be the most potent SARS-CoV- 3CL(pro) inhibitor. Kinetic studies showed that both alkylated chalcones were competitive inhibitors (Park O. K. et al., 2016). Since xanthoangelol E was also found to inhibit SARS-CoV- PLpro (Park O. K. et al., 2016) it could be a promising candidate in the therapeutic approach against COVID-19.

### Phlorotannins From *Ecklonia cava* (Algae)

Park et al. (2013) isolated nine phlorotannins from the ethanolic extract of brown Alga *Ecklonia cava*. These phlorotannins were assessed regarding their inhibitory effects towards SARS-CoV- 3CL(pro) using a cell-free based assay. Eight phlorotannins (triphloretol A, eckol, dioxinodehydroeckol, 2-phloroeckol, 7-phloroeckol, fucodiphloroethol G, dieckol, and phlorofucofuroeckol A) were shown to be competitive inhibitors of SARS-CoV- 3CL(pro) in a dose dependent manner. IC_50_ ranged from 2.7 ± 0.6 (dieckol) to 164.7 ± 10.8 µM (triphloretol A). Moreover, six phlorotannins (dioxinodehydroeckol, 2-phloroeckol, 7-phloroeckol, fucodiphloroethol G, dieckol, and phlorofucofuroeckol A) resulted in a significant micromolar dose-dependent inhibition of SARS-CoV-3CL(pro) cis-cleavage activity. Of the tested molecules, diekcol (possessing two eckol groups linked by a diphenyl ether) exhibited the best inhibitory effect of SARS-CoV-3CL(pro). Molecular docking studies corroborated this result since diekcol possessed the lowest binding energy (11.51 kcal/mol) towards SARS-CoV-3CL(pro). Diekcol was shown to form strong H bonds to the catalytic dyad (Cys145 and His41). Nevertheless, the bioavailability of phlorotannins and the inter-individual differences regarding their metabolization is still a substantial limitation to validate their usefulness. Gut microbiota composition seems to play a critical role in determining their health benefits (Corona et al., 2016). Moreover, the complexity of their structures due to the diversity of structural linkages and the different structural and conformational isomers for the same molecular weight, in addition to the absence of analytical standards and the lack of clear relationship between their structure and bioactivity may be another limitation to their clinical use (Li et al., 2017).

### Tanshinones From *Salvia miltiorrhiza* Bunge

Park O. K. et al. (2012) investigated the inhibitory potential of *Salvia miltiorrhiza* Bunge towards SARS-CoV-3CL(pro). They found that *Salvia miltiorrhiza* Bunge ethanolic extract (30 µg/ml) resulted in 60% inhibition of SARS-CoV-3CL(pro). Furthermore, they demonstrated that six tanshinones of the plant (lipophilic fraction) exerted marked inhibition of SARS-CoV-3CL(pro) in a dose- but not–time- dependent manner. IC_50_ was estimated at 14.4–89.1 µM. Dihydrotanshinone I exhibited the most important inhibitory effect with an IC_50_ of 14.4 ± 0.7 µM. With regard to the kinetic mechanism of SARS-CoV-3CL(pro) inhibition, *Salvia miltiorrhiza* Bunge tanshinones were found to be noncompetitive inhibitors.

### Biflavonoids From *Torreya nucifera* (L.) Siebold & Zucc.

Four biflavonoids (amentoflavone, bilobetin, ginkgetin, and sciadopitysin) were isolated from the ethanol extract of *Torreya nucifera* (L.) Siebold & Zucc. leaves and evaluated for their SARS-CoV-3CL(pro) inhibitory effect by using a FRET method. All biflavonoids exhibited a marked inhibitory effect of SARS-CoV-3CL(pro) with IC_50_ of 8.3–72.3 µM. This inhibitory effect was stronger than that of eight diterpenoids isolated from the *T. nucifera* extract (IC_50_: 49.6–283.5 µM). Amentoflavone exerted the most important inhibitory activity since it possessed the lowest IC_50_ (8.3 ± 1.2 µM). Moreover, its inhibitory potential was more important than that of apigenin (IC_50_ = 280.8 ± 21.4 µM), quercetin (IC_50_ = 23.8 ± 1.9 µM) and luteolin (IC_50_ = 20.0 ± 2.2 µM). Molecular docking demonstrated that amentoflavone showed a good affinity with SARS-CoV-3CL(pro) and formed strong hydrogen bonds. An apigenin moiety at position C-30 of flavones was suggested to be responsible for a better inhibitory effect (Ryu et al., 2010).

### Flavonoids

Seven flavonoids (Quercetin, Puerarin, Daidzein, gallocatechin gallate, epigallocatechin gallate, epigallocatechin, ampelopsi) were evaluated for their inhibitory effects of SARS-CoV-3CL(pro) expressed in *Pichia pastoris* GS115. At 200 µM, gallocatechin gallate, epigallocatechin gallate, and quercetin were able to inhibit the SARS-CoV-3CL(pro) activity by 91, 85, and 82%, respectively. Gallocatechin gallate was found to be a competitive inhibitor of SARS-CoV-3CL(pro) with IC_50_ of almost 47µM. Molecular docking studies confirmed the important inhibitory potential of gallocatechin gallate owing to the hydrophobic and H-bonds interaction formed with the active site of SARS-CoV-3CL(pro) (Nguyen et al., 2012). Nonetheless, it is difficult to predict how these H-bonds may contribute to the biological functions of the supposed active molecules (Chen et al., 2016). Furthermore, the strength of the H-bonds is not evaluated in this study which could limit the selectivity of the tested molecules since an important number of weak H-bonds often increase their affinity and therefore their interaction with off-target proteins.

Jo et al. (2020) evaluated the inhibitory effect of 64 flavonoids towards SARS-CoV-3CL(pro) using the FRET method. They found that at 20 µM, rhoifolin, herbacetin, and pectolinarin possessed the higher inhibitory effect, with IC50 values of 27.45, 33.17, and 37.78 μM, respectively. In addition, molecular docking revealed that flavonoids possessed an important binding affinity for SARS-CoV-3CL(pro) owing to their hydrophobic aromatic rings and hydrophilic hydroxyl groups.

**Figure 6:**
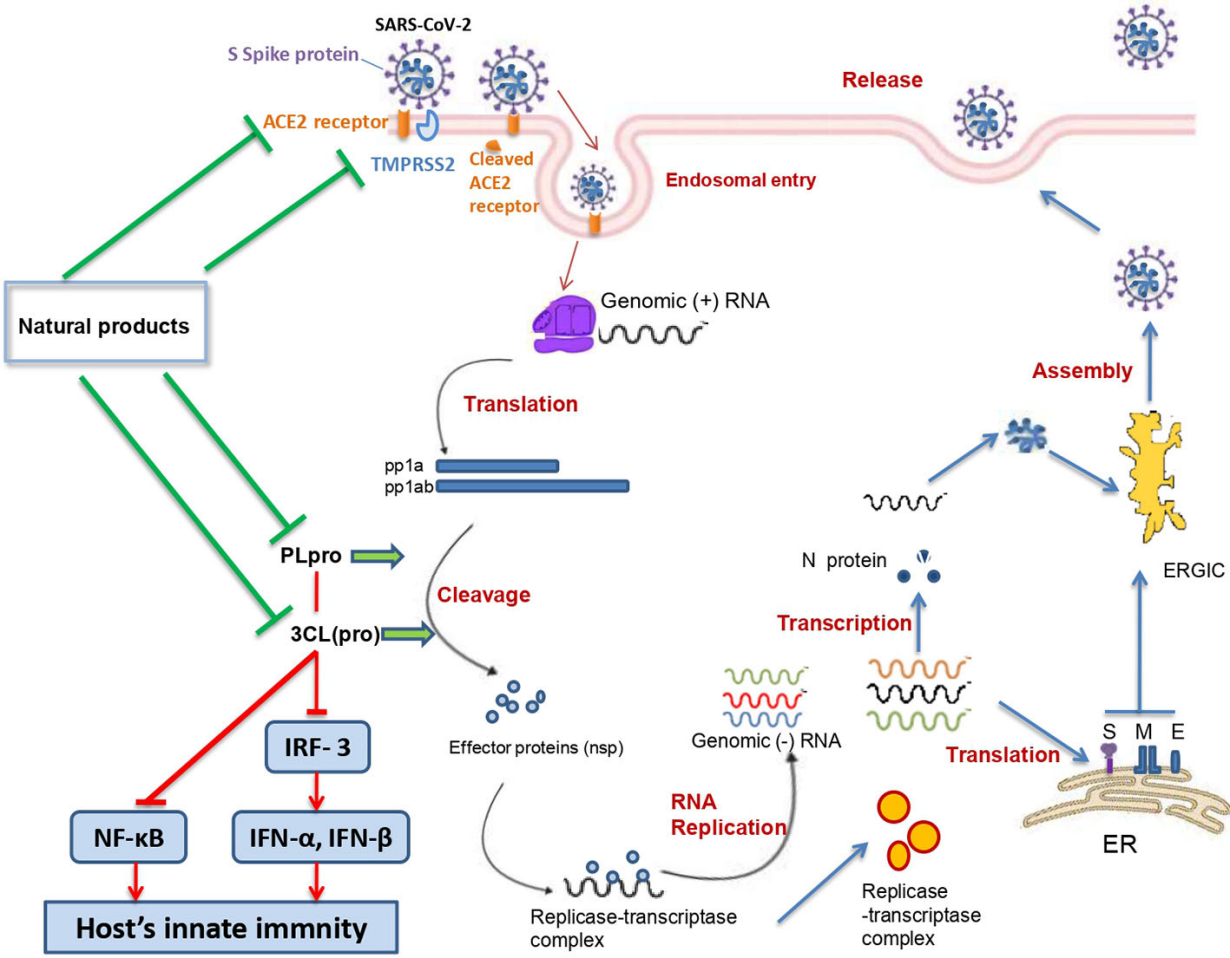
Summary of possible anti SARS-CoV 2 actions of natural products.

**Figure 6** summarizes the possible anti-SARS-CoV 2 actions of natural products.

## Critical Considerations

In the present review, 15 *in vitro* studies were included. They were from Korea (9/15), Taiwan (3/15), China (2/15), and Iran (1/15). Most of the studies (10/15) were published between 2011 and 2018 whereas only two studies (Runfeng et al., 2020; Jo et al., 2020) were published in 2020 and one study (Weng et al., 2019) in 2019. Of them, only one study (Runfeng et al., 2020) investigated the antiviral activity against SARS-CoV-2, and another (Weng et al., 2019) on the human coronavirus NL63, whereas the other studies were not cell-based. Three studies used plant extracts and one study (Runfeng et al., 2020), a herbal mixture of 11 Chinese plants. Ethanol, methanol, and water were the solvents used to prepare the plant extracts included in these studies. Most of the studies were conducted with isolated natural compounds belonging to different phytochemical classes. Phenolic compounds were the most frequently reported.

When studies were analyzed using the best practice recommendations in phytopharmacological research (Heinrich et al., 2020), several concerns were detected especially regarding reporting data and outcomes. A large number of natural products reported in this review such as phenolic acids, quercetin, or kaempferol belong to phytochemical classes known to possess a broad spectrum of biological activities both *in vitro* and *in vivo*. Therefore, most of the studies failed to demonstrate the specificity of such products. In addition, all the included studies did not consider the drugability of the “active” compounds or extracts and presented an insufficient interpretation of the obtained data. Likewise, all the studies presented limitations regarding concepts and methods and also in the development of the project. In fact, all the studies investigating herbal extracts and isolated compounds did not consider the sustainable sourcing of the species nor the registration standards of both compounds or accepted plants’ names. Likewise, the included studies presented serious bias concerning the dose range and the toxic doses. On the other hand, eight studies did not report the use of control, whereas the other studies did not justify the choice of the positive controls used for comparison. This could be considered as a risk of bias resulting in possible limitations in the methodology of the studies. Besides, the lack of full taxonomic validity has been detected in all these studies.

It has been reported that IC_50_ of chloroquine inhibition of SARS-CoV-2 replication was found to be 1.13 to 5.47 µM (Smit et al., 2020) whereas that of SARS-CoV was 8.8 µM (Keyaerts et al., 2004). On the other hand, IC_50_ of ivermectin was found to be ~2 μM (Caly et al., 2020). Therefore, molecules with IC_50_ ranging from 0 to 10 µM have been considered as possible active molecules against SARS-CoV-2. Accordingly, few natural molecules reported in the included studies could be considered active against SARS-CoV-2 including amentoflavone, dieckol, hirsutenone, cryptotanshinone, xanthoangelol E, tomentin E, psoralidin, scutellarein, myricetin, and caffeic acid. Nevertheless, most of these active molecules are phenolic compounds characterized by a low bioavailability and rapid elimination (Górniak et al., 2019) which could compromise their clinical usefulness in the context of COVID-19.

Despite the promising possible anti-SARS-CoV-2 effects exhibited by both plant extracts and natural molecules, several limitations should be considered. Overall, due to the recent outbreak, the clinical usefulness of these products needs to be demonstrated since the current data are still immature, and no final conclusions have been validated. As shown in **Table 2**, for some species, there was no relationship between the traditional ethnopharmacological uses and the anti-SARS-CoV-2 effects. In spite of that, these plants are currently used to treat or manage symptoms reported in SARS-CoV-2 disease such as fever, inflammations, or cardiovascular and circulatory disorders. Moreover, efficacy and safety of the active natural products should be further studied *in vivo* and clinically validated in COVID-19 patients. Importantly, bioavailability, modes of administration, safe doses, time of exposure, pharmacokinetic profile, the health of the patients’ digestive system, and disease stage are to be considered in the evaluation of the beneficial effects of natural products against SARS-CoV-2. On the other hand, further studies are needed to clarify the mechanisms and pathways targeted by such products, which will help to improve their clinical usefulness. Assessing the effects of combinations of active natural products with validated antiviral drugs could be a promising alternative to explore.

## Conclusion

Medicinal plants and natural products are still considered promising alternatives to prevent or treat several diseases. Since the outbreak of the COVID-19 pandemic in December 2019, various traditional herbal medicines have been used and resulted in positive health effects among COVID-19 patients, mainly in China. In the present review, we have discussed the possible potential uses of medicinal plants and/or natural products to prevent or even treat COVID-19. Although the studies evaluating the anti-SARS-CoV-2 effects of medicinal plants are still insufficient and relatively immature, some natural products with IC_50_ below 10 µM could be considered as promising anti-SARS-CoV-2 agents since they were able to block its life-cycle related proteins such as the cellular receptor ACE2, papain-like or chymotrypsin-like proteinases. Nevertheless, several limitations have been detected in relation to the specificity of the action exerted by such products, sustainable sourcing of the species, doses range used, or the use of appropriate controls.

While available studies offer several indications that these plant-derived products may help in fighting COVID-19, further studies should be carried out to evaluate the clinical usefulness of such products against COVID-19 infection. Furthermore, the bioavailability of natural products with possible anti-SARS-CoV-2 effects such as tannins should be considered besides the need for clinical validation of their usefulness and safety. The herbal mixtures, medicinal plants, or natural products with possible anti-SARS-CoV-2 effects must be evaluated through prospective and interventional studies. A combination of natural products or herbal mixtures with validated anti-COVID-19 drugs may constitute a promising preventive and therapeutic alternative to be assessed.

## Author Contributions

BB and AP contributed equally to the study. All authors contributed to the article and approved the submitted version.

## Conflict of Interest

The authors declare that the research was conducted in the absence of any commercial or financial relationships that could be construed as a potential conflict of interest.
